# Supplementary material for: Complex‐centric proteome profiling by SEC‐SWATH‐MS
Source: Mol Syst Biol. 2019 Jan 14;15(1):e8438. doi: 10.15252/msb.20188438 (PMC6346213; doi:10.15252/msb.20188438)
Supplement: Supplementary file 7 — Dataset EV6 [file MSB-15-e8438-s007.zip › feature_plots_bioplex/Q6DKI7.pdf]

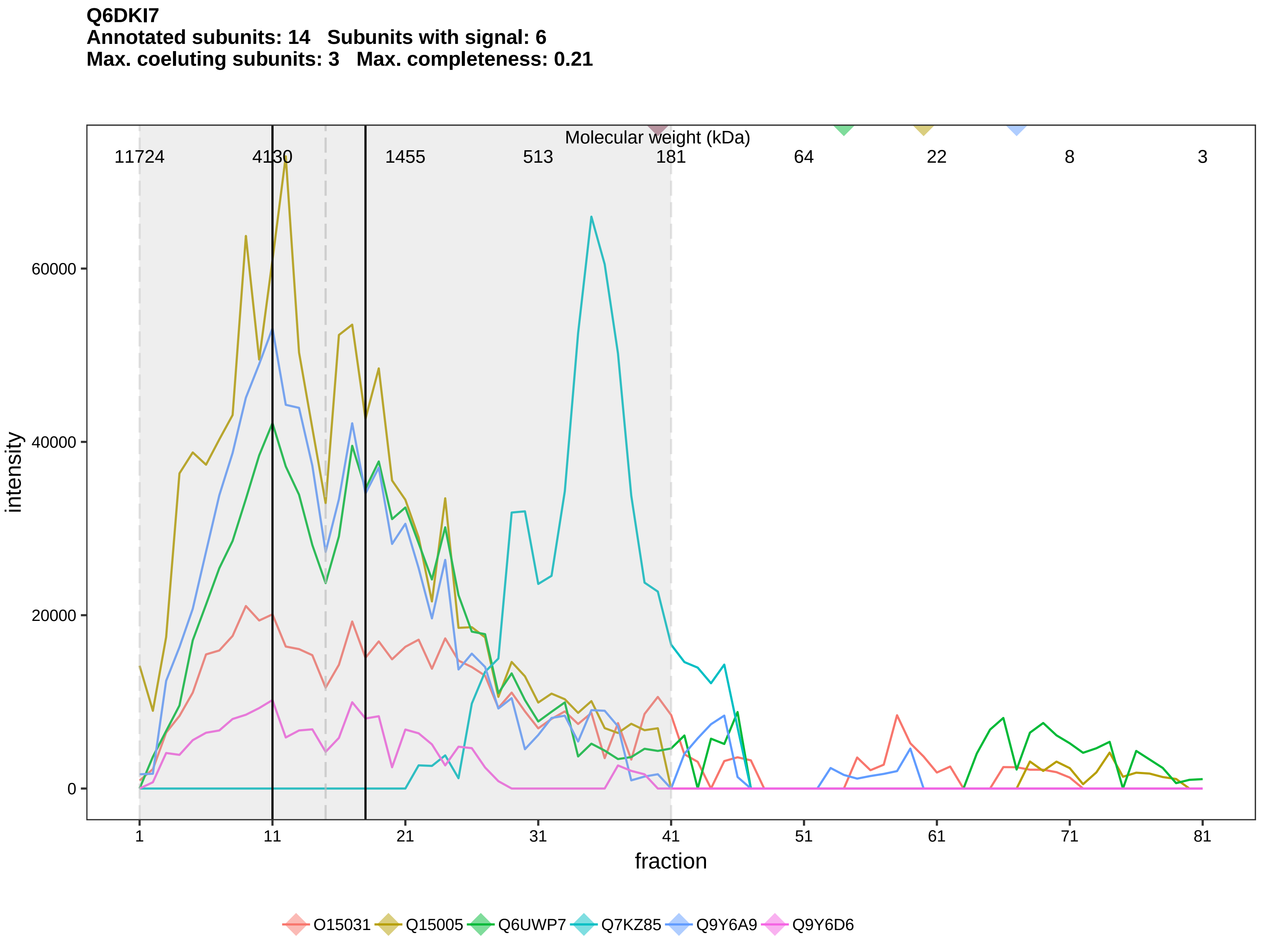

Q6DKI7
Annotated subunits: 14   Subunits with signal: 6
Max. coeluting subunits: 3   Max. completeness: 0.21
Molecular weight (kDa)
11724
4130
1455
513
181
64
22
8
3
60000
40000
20000
0
intensity
1
11
21
31
41
51
61
71
81
fraction
O15031
Q15005
Q6UWP7
Q7KZ85
Q9Y6A9
Q9Y6D6
